# Primary macrophages and J774 cells respond differently to infection with *Mycobacterium tuberculosis*

**DOI:** 10.1038/srep42225

**Published:** 2017-02-08

**Authors:** Nuria Andreu, Jody Phelan, Paola F. de Sessions, Jacqueline M. Cliff, Taane G. Clark, Martin L. Hibberd

**Affiliations:** 1 Pathogen Molecular Biology Department, London School of Hygiene and Tropical Medicine, London, UK; 2 Genome Institute of Singapore, A*STAR, Singapore; 3 TB Centre and Department of Immunology and Infection, London School of Hygiene and Tropical Medicine, London, UK; 4 Department of Infectious Disease Epidemiology, London School of Hygiene and Tropical Medicine, London, UK

## Abstract

Macrophages play an essential role in the early immune response to *Mycobacterium tuberculosis* and are the cell type preferentially infected *in vivo*. Primary macrophages and macrophage-like cell lines are commonly used as infection models, although the physiological relevance of cell lines, particularly for host-pathogen interaction studies, is debatable. Here we use high-throughput RNA-sequencing to analyse transcriptome dynamics of two macrophage models in response to *M. tuberculosis* infection. Specifically, we study the early response of bone marrow-derived mouse macrophages and cell line J774 to infection with live and γ-irradiated (killed) *M. tuberculosis*. We show that infection with live bacilli specifically alters the expression of host genes such as *Rsad2, Ifit1/2/3* and *Rig-I*, whose potential roles in resistance to *M. tuberculosis* infection have not yet been investigated. In addition, the response of primary macrophages is faster and more intense than that of J774 cells in terms of number of differentially expressed genes and magnitude of induction/repression. Our results point to potentially novel processes leading to immune containment early during *M. tuberculosis* infection, and support the idea that important differences exist between primary macrophages and cell lines, which should be taken into account when choosing a macrophage model to study host-pathogen interactions.

*Mycobacterium tuberculosis* infection occurs by inhalation of bacilli-containing aerosols produced by patients with active pulmonary disease. In the lung, the first host cells that the bacilli encounter are the alveolar macrophages, which can identify and phagocytise *M. tuberculosis* via specific receptors such as toll-like receptors (TLRs), scavenger receptors or mannose receptor^1^. These interactions lead to production of various antimicrobial peptides, cytokines and chemokines^2^. If the pathogen is not eliminated within the alveolar macrophages, other cell types such as monocytes, dendritic cells and neutrophils are recruited and infected, leading to granuloma formation^3^. Once the adaptive immunity ensues, CD4^+^ T-cells arrive to the lung secreting IFN-γ that activates macrophages for efficient *M. tuberculosis* killing. Therefore, macrophages are both key effector cells in mycobacterial killing and a niche for bacterial multiplication. Understanding the mechanisms of *M. tuberculosis*-macrophage interactions is key to identify pathways central to both bacterial virulence and host immunity.

Several macrophage models have been used to study *M. tuberculosis* infection. These include primary macrophages, mainly human monocyte-derived macrophages (hMDMs) or mouse bone marrow-derived macrophages (BMDMs); and cell lines, such as the human THP-1 and U937, or the murine J774 and RAW 264.7^4^,^5^. BMDMs are a useful model because they can be easily cultured and expanded to large numbers. Besides, BMDMs isolated from knockout mice enable the study of the role of specific genes/pathways during *M. tuberculosis* infection. hMDMs are a more physiologically relevant model, since they are isolated from the natural host. Additionally, hMDMs are relatively easy to obtain and differentiate *in vitro* from human blood monocytes. However, the use of human biological samples entails ethical issues, and the genetic variation that underlies the use of human donors complicates result interpretation. The use of cell lines, on the other hand, has several advantages, related mainly to the fact that they are homogenous, and very easy to propagate and maintain in the laboratory. However, cell lines derive from transformed or immortalized cells that have acquired genetic and phenotypic differences in comparison with their primary counterparts^6^,^7^,^8^. Furthermore, cell lines have a tendency to be genetically unstable, with hybrid phenotypes and possibly atypical signalling mechanisms. Therefore, the question remains on whether cell lines are physiologically relevant systems, particularly for host-pathogen interaction studies.

Previous comparisons of macrophage infection models for *M. tuberculosis* have focused on certain phenotypic traits such as intracellular growth, cytokine production, or phagosome characteristics^5^,^9^,^10^. Jordao and collaborators found that *Mycobacterium bovis* grows more efficiently in hMDMs than in J774 over a 7-day time course, even though both types of macrophages can restrict growth during the first 24 hours of infection through a mechanism involving phagosome acidification and nitric oxide production^9^. Mendoza and collaborators compared *M. tuberculosis, M. bovis* and *M. bovis* BCG infection of hMDMs, THP-1 and U937, and found that U937 has a lower phagocytic capacity than the other two. They also compared production of nitrite, TNF-α and IL-12 in resting and IFN-γ activated macrophages, and found that THP-1 resembles more closely hMDMs^10^.

Here we focus on two mouse macrophage models that are widely used to study *M. tuberculosis* infection: BMDMs (primary macrophages) and J774A.1 (a cell line, hereafter referred to as “J774” for simplicity). We conduct an in depth analysis of their transcriptomic response to infection with live *M. tuberculosis* and stimulation with γ-irradiated (killed) *M. tuberculosis*. We find that BMDMs respond very strongly to infection whereas the response of J774 is delayed and more discreet both in terms of number of differentially expressed genes and magnitude of induction/repression. Furthermore, we identify macrophage genes whose possible function in resistance to *M. tuberculosis* infection has not yet been investigated.

## Results

### Global transcriptome profiles

BMDMs and J774 macrophages were either infected with live *M. tuberculosis* H37Rv or stimulated with γ-irradiated (killed) *M. tuberculosis* H37Rv for 4 hours. Total RNA was isolated at 4 and 24 hours post-infection (hpi), and CFUs were determined by plating dilutions of lysed macrophages. At 24 hpi, a 0.34 log and a 0.2 log decrease in CFUs was observed in BMDMs and J774, respectively (see Supplementary Fig. S1). Total RNA was used to construct individually barcoded, strand-specific RNA-seq libraries after depletion of ribosomal and mitochondrial RNA, and the libraries were sequenced on an Illumina^®^ HiSeq™ 2000 apparatus.

To investigate general trends in the data, principal component analysis (PCA) was carried out (see Supplementary Fig. S2). PCA confirmed no outlier samples and showed separate grouping of uninfected macrophages (controls), and macrophages infected with live bacteria or stimulated with dead bacteria. The transcriptome profiles of BMDMs infected with live bacteria or stimulated with dead bacteria were similar to each other at 4 hpi and became more distinct (from each other and especially from the 4-hpi samples) at 24 hpi. For the J774 cells, PCA showed grouping of the transcriptomes of cells infected with live bacteria or stimulated with dead bacteria at 4 hpi and those of cells stimulated with dead bacteria at 24 hpi; that is, in the case of J774 cells, the 24-hpi transcriptome of cells stimulated with dead bacteria was not clearly different from the 4-hpi transcriptomes.

In addition, the transcriptome of BMDMs uninfected controls (but not that of J774 uninfected cells) changed considerably from 4 hpi to 24 hpi (Supplementary Fig. S2, and Supplementary Data S1). That is, considerable changes occurred in the transcriptome of uninfected BMDMs while they were maintained in culture, emphasising the need to process a matched non-infected control for each time point when using primary macrophages. These differences in the transcriptome of *in vitro* grown primary macrophages over time had been previously observed by others both with murine macrophages^11^ and human macrophages^12^.

### Differences between the transcriptional responses of BMDMs and J774 cells

**Figure 1:**
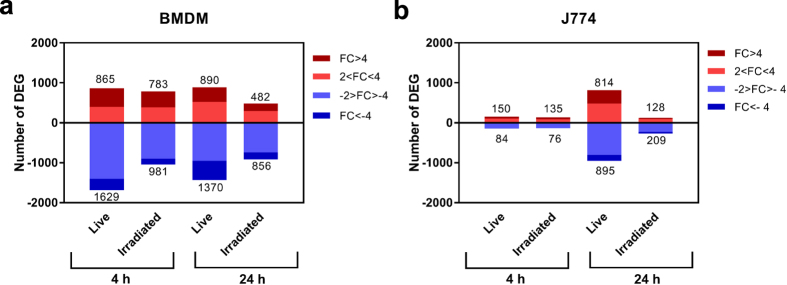
Number of differentially expressed genes (DEGs). (**a**) In BMDMs, and (**b**) in J774 infected with live *M. tuberculosis* or stimulated with γ-irradiated *M. tuberculosis*, relative to their time-matched uninfected controls, at 4 and 24 hpi (FDR < 0.05). The colour shading indicates the fold change in gene expression. The number of DEGs is indicated.

**Figure 2:**
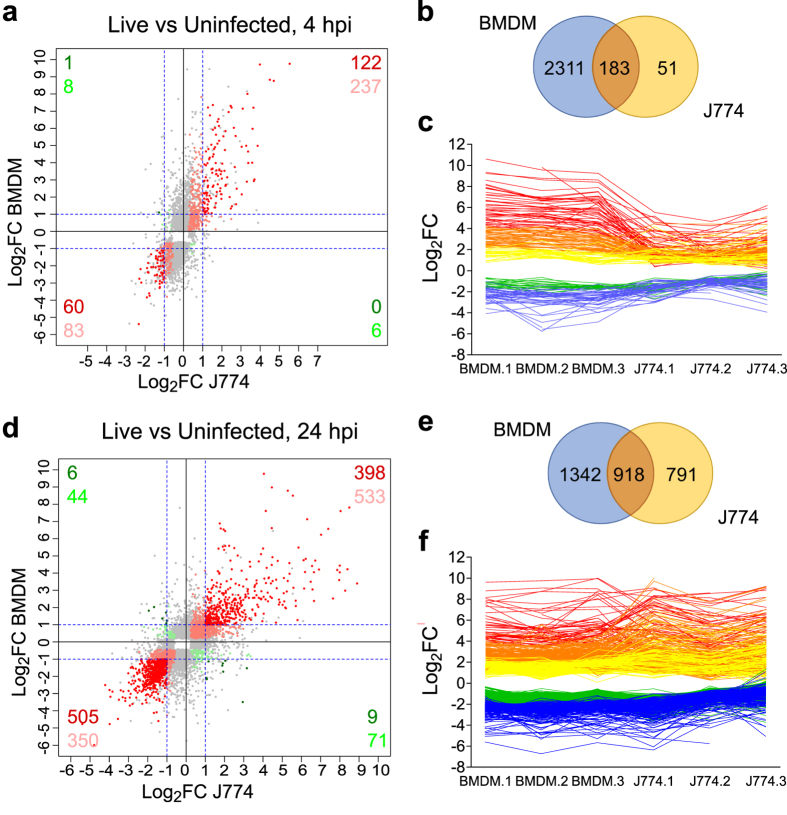
Comparison of the response of BMDMs and J774 to infection with live *M. tuberculosis*. Scatterplots comparing the Log_2_ fold change (FC) of all the differentially expressed genes (DEGs) in BMDMs and J774 at 4 hpi (**a**) and 24 hpi (**d**). Grey dots represent genes differentially expressed in one macrophage type only. Points in shades of red represent genes differentially expressed in both data sets in the same direction. Points in shades of green represent genes differentially expressed in opposite direction. Those showing FC < |2| in either/both condition(s) are shown in light colour and those with FC > |2| in both conditions are shown in dark. The number of genes are indicated for each quadrant. (**b)** and (**e)** Venn diagrams comparing DEGs in BMDMs and J774 at 4 hpi and 24 hpi, respectively. (**c)** and (**f)** Line graphs showing the variation of the Log_2_FC for each DEG in common between BMDMs and J774 at 4 hpi (**c**) and 24 hpi (**f**). Lines are coloured according to the Log_2_FC in BMDMs (Log_2_FC > 4 red, 2 < Log_2_FC < 4 orange, 1 < Log_2_FC < 2 yellow, −1 > Log_2_FC > −2 green, −2 > Log_2_FC blue).

Differential expression analysis demonstrated a clear response of *M. tuberculosis*-infected BMDMs as early as 4 hpi. A total of 2,494 significantly differentially expressed genes (DEGs) with an absolute fold change (FC) greater than 2 (|FC|>2 or |Log_2_FC|>1) were detected at 4 hpi in BMDMs infected with live bacteria compared to uninfected BMDMs (FDR < 0.05) (Fig. 1a, and Supplementary Data S2). By contrast, only 234 DEGs were detected for J774 when comparing uninfected vs infected with live bacteria (FDR < 0.05, |FC|>2, Fig. 1b, and Supplementary Data S2). Of these 234 DEGs, 183 were also differentially expressed in BMDMs (Fig. 2a and b, and Supplementary Data S3), representing 78% and 7.4% of the DEGs in J774 and BMDMs, respectively. Furthermore, 182 of the 183 common DEGs were differentially expressed in the same direction, with 122 upregulated (top right quadrant of the scatter plot in Fig. 2a), and 60 downregulated (bottom left quadrant, Fig. 2a). However, the absolute fold change in expression of 136 (74.3%) of the 183 common DEGs was higher in BMDMs than in J774 (Fig. 2c). This was a general trend, with a Log_2_FC range between −5.39 and +9.76 in BMDMs, whereas in J774 it was narrower, between −2.33 and +5.54.

The number of DEGs in J774 (but not in BMDMs) increased considerably from 4 to 24 hpi, but was still lower than the number of DEGs in BMDMs (Fig. 1, and Supplementary Data S2). 918 genes with a |FC|>2 were differentially expressed at 24 hpi in both BMDMs and J774 cells (Fig. 2d and e, and Supplementary Data S3). These represent 41% and 54% of the total DEGs at 24 hpi, respectively. Of these 918 common DEGs, 903 were differentially expressed in the same direction, with 398 upregulated (top right quadrant of the scatter plot), and 505 downregulated (bottom left quadrant, Fig. 2d). At 24 hpi, the level of response was more similar between the two types of cells, with only 55% of the common DEGs presenting a Log_2_FC significantly different (Fig. 2f). Overall, the global level of induction/repression was similar at 24 hpi in both types of macrophages ranging from −6.00 to +9.77 in BMDMs, and −5.41 to +9.84 in J774. Of note, 104 of the common DEGs detected at 4 hpi were also differentially expressed in both cell types at 24 hpi, including most of the top differentially expressed ones.

### Core response of murine macrophages to infection with live *M. tuberculosis*

A more detailed examination of the genes differentially expressed in both J774 and BMDMs highlighted many genes previously described as important during *M. tuberculosis* infection (Supplementary Data S3). These include receptors for the recognition and phagocytosis of *M. tuberculosis*, such as the C-type lectin receptor Mincle (*Clec4e*) and the class A scavenger receptor *MARCO*, both of which were induced; the mannose receptor (*Mcr1*), that was repressed; and the cytosolic receptors *Nod1* and *Nod2*, and *Tlr2*, induced. The products of other shared induced genes are involved in the activation and recruitment of antimicrobial mechanisms, like the CD40 receptor that activates antimicrobial mechanisms in infected macrophages upon interaction with ligand CD40L in T-cells^13^; several IFN-inducible GTPases such as *Gbp6, Gbp7, Irgm1 (Lrg-47*) and *Irgm2*, which confer cell-autonomous immunity to mycobacterial infection^14^,^15^; *Isg15*, which induces IFNγ-production in lymphocytes^16^; *Irg1*, with bactericidal properties against *M. tuberculosis* through various mechanisms including inhibition of its isocitrate lyase^17^,^18^,^19^; *Il1b*, which stimulates antimycobacterial immunity in macrophages^20^,^21^, and caspase-1 (*Casp1*), required for processing of pro-IL-1β into its active form^22^. *Mmp9* and *Ccl5 (Rantes*), associated with recruitment of monocytes and macrophages during granuloma formation^23^,^24^, and the neutrophil and lymphocyte-recruiting chemokines *Cxcl2, Cxcl10 and Cxcl11* were also induced; as were the pro-apoptosis genes *Ptgs2 (Cox2*) and *Ptges (Pges*), while the pro-necrosis gene *Alox5* was repressed.

Several interferon-stimulated genes (ISGs) were also highly induced, such as *Rsad2 (Viperin), Mx1, Mx2, Ifit1, Ifit2, Ifit3, Ifi205, Apol9a,* and *Apol9b*, all with known antimicrobial functions^25^,^26^,^27^ but without a known function during *M. tuberculosis* infection. Interestingly, the gene encoding RIG-I (*Ddx58*) was also induced. RIG-I is a pattern recognition receptor involved in viral RNA recognition, critical for the host antiviral response. The top downregulated genes included *Rtn4rl1*; the apoptosis activator *Bmg*; the receptor *Epha2*, whose absence is associated with a reduced bacterial load during the chronic phase of *M. tuberculosis* infection in mice^28^*; Gpr34,* whose deficiency in mice has been associated with an altered immune response^29^,^30^; the chemokine *Ccl24*; and the scavenger receptor *Fcrls*.

### Pathway analysis of DEGs in macrophages infected with live *M. tuberculosis*

To gain insight into the cellular and molecular functions of the *M. tuberculosis*-induced host genes, DEGs in macrophages infected with live bacteria were subjected to pathway analysis using QIAGEN’s Ingenuity^®^ Pathway Analysis (IPA^®^). A full list of all the enriched pathways for each condition can be found online in the Supplementary Data S4. Comparison analysis was used to visualise the results across all four conditions.

**Figure 3:**
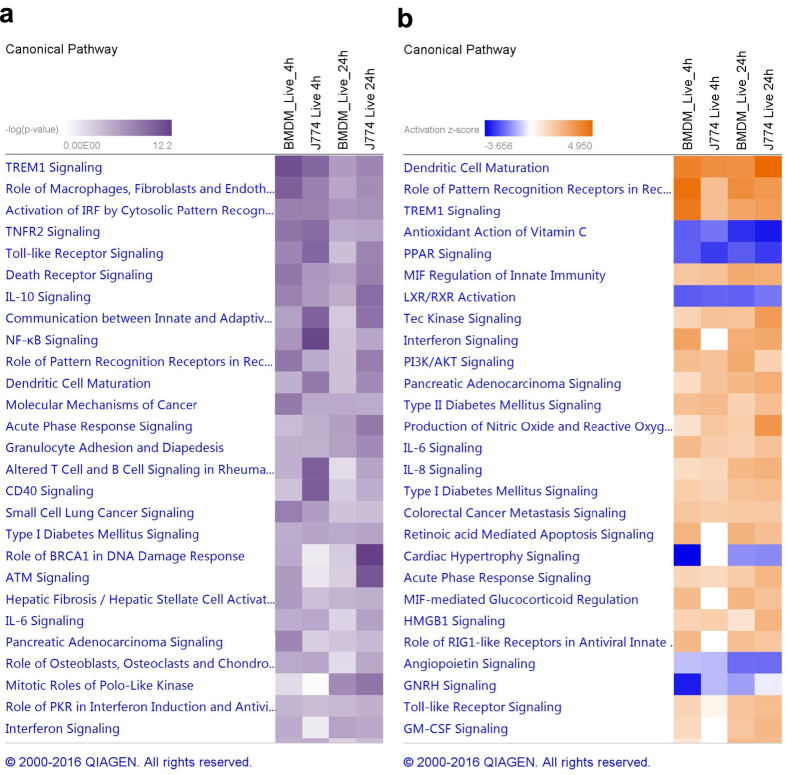
Top enriched pathways in macrophages infected with live *M. tuberculosis*. Data were analysed through the use of QIAGEN’s Ingenuity^®^ Pathway Analysis (IPA^®^, QIAGEN Redwood City, www.qiagen.com/ingenuity). Pathways are ranked according to (**a**) the enrichment score (Fisher’s exact test *P*-value), and (**b**) the *Z*-score that predicts activation/repression.

The top enriched pathways are shown in Fig. 3. Pathways are ranked according to the enrichment score (Fisher’s exact test *P*-value, Fig. 3a), and to the *Z*-score which predicts activation or inhibition of the pathways by comparing observed and predicted regulation patterns (Fig. 3b). The same pathways were enriched in BMDMs and J774 although with different scores. For most pathways, the enrichment score and the *Z*-score was higher in BMDMs than in J774, particularly at 4 hpi, reflecting the stronger response in this type of macrophages early after the infection (Fig. 3).

TREM1 (triggering receptor expressed on myeloid cells 1) was one of the most significantly enriched pathways, with a high activation score in all conditions. TREM1 is a DAP12-associated receptor that plays an essential role in innate immunity by fine-tuning the inflammatory response^31^. In our dataset, many of the genes encoding cytokines and chemokines whose expression and secretion is induced by TREM1 activation, as well as several genes encoding cell surface proteins, were upregulated (see Supplementary Fig. S3). In addition to regulating the innate immune response, TREM1 modulates the adaptive immune response by inhibiting the regulators SIGIRR, which downregulates the Th1 response, and ST2, which enhances the Th2 response^32^. Both SIGIRR and ST2 were downregulated in our dataset.

Several pathways involved in bacterial recognition by host cells were also activated such as “Activation of IRF by cytosolic pattern recognition receptors”, “Role of RIG-1 like receptors in antiviral innate immunity”, “Toll-like receptor signalling”, or the more general pathway “Role of pattern recognition receptors in recognition of bacteria and virus” which includes TLR, NLR, RIG and complement receptors (Fig. 3).

Only a few pathways had a negative *Z*-score (inhibition), including PPAR signalling, LXR/RXR activation, and antioxidant action of vitamin C (Fig. 3b). Of these, PPAR signalling had the highest enrichment score. The PPAR (proliferator-activated receptor) family are ligand-activated transcriptional regulators activated by fatty acids and their derivatives. They regulate the expression of genes involved in lipid metabolism, and their function is supressed by cytokines such as IL-1 and TNF-α.

### Cell type-specific response to *M. tuberculosis* infection

A full list of the genes differentially expressed exclusively in one of the two macrophage models at a particular time point can be found online (Supplementary Data S3). Some of the genes induced exclusively in BMDMs included the chemokines *Cxcl1, Cxcl3* and *Cxcl9*, the cytokines *Il1a, Il6*, and *Il12b*; *Rasgrp1*, which participates in T cell activation; *Mmp14*, which regulates monocyte migration and collagen destruction in tuberculosis^33^; *Serpinb2*, also known as plasminogen activator inhibitor type 2 (*Pai-2*), a major product of activated monocytes/macrophages which has been linked to regulation of Th1 responses^34^; and *Fpr2*, a recently described pathogen recognition receptor that recognizes bacterial signal peptides and triggers classical innate immune responses, such as intracellular Ca^2+^ mobilization, generation of reactive oxygen species, release of metallopeptidase, and chemotactic cell migration^35^. *Hes1*, a Notch target gene that suppress TLR-triggered pro-inflammatory cytokine production^36^, was one of the most downregulated genes in BMDMs.

Among the top induced genes solely in J774 there was *Nos2*, highly induced at 24 hpi; *Rab15*, involved in endocytic recycling^37^,^38^; the gene encoding for the laminin subunit gamma-2 (*Lamc2*); *Kdm6b* also known as *Jmjd3*, a lysine-specific demethylase that modulates pro-inflammatory responses in macrophages^39^ and has been recently implicated in foamy macrophage formation during mycobacterial infection infection^40^; the IFN-inducible GTPase *Gbp1*; and *Il23a*, required for long term control of *M. tuberculosis*^41^ (Supplementary Data S3). *Tlr5* and *Tlr8* were some of the downregulated genes exclusively in J774, together with *Il10* that was repressed at 24 hpi in J774 while it was induced in BMDMs at 4 hpi.

Pathway analysis did not reveal any cell-specific pathways, although at 4 hpi some pathways were enriched exclusively in BMDMs, which is not unexpected given the modest response exhibited by J774 cells at this early time point (Fig. 3). Some of the pathways induced in BMDMs at 4 hpi but not in J774 until 24 hpi were “Interferon signalling” and “Role of RIG1-like receptors in antiviral innate immunity”.

### Immune response of macrophages to stimulation with γ-irradiated *M. tuberculosish*

To study if the differences detected between both cell types were due to differences in the response to either phagocytosis or infection, the transcriptome of macrophages stimulated with dead (γ-irradiated) *M. tuberculosis* was also analysed by RNA-seq. Similar to what was observed when infecting with live bacteria, differential expression analysis of macrophages stimulated with dead bacteria compared to uninfected macrophages at 4 hpi showed a stronger response in BMDMs (1,764 DEGs) than in J774 (211 DEGs) (Fig. 1). At 24 hpi the number of DEGs slightly decreased in BMDMs (1,338 DEGs) and increased in J774 (337 DEGs). Moreover, the magnitude of regulation in macrophages infected with live bacteria and stimulated with dead bacteria was similar in BMDMs at both time points, and in J774 at 4 hpi, but lower in J774 stimulated with dead bacteria at 24 hpi. Specifically, the Log_2_FC in gene expression ranged from −5.73 to +9.71 and from −5.00 to +9.98 in BMDMs at 4 hpi and 24 hpi, respectively, and from −3.28 to +5.66 and −5.15 to +5.73 in J774. A full list of all the DEGs (|FC|>2) can be found online in the Supplementary Data S2.

**Figure 4:**
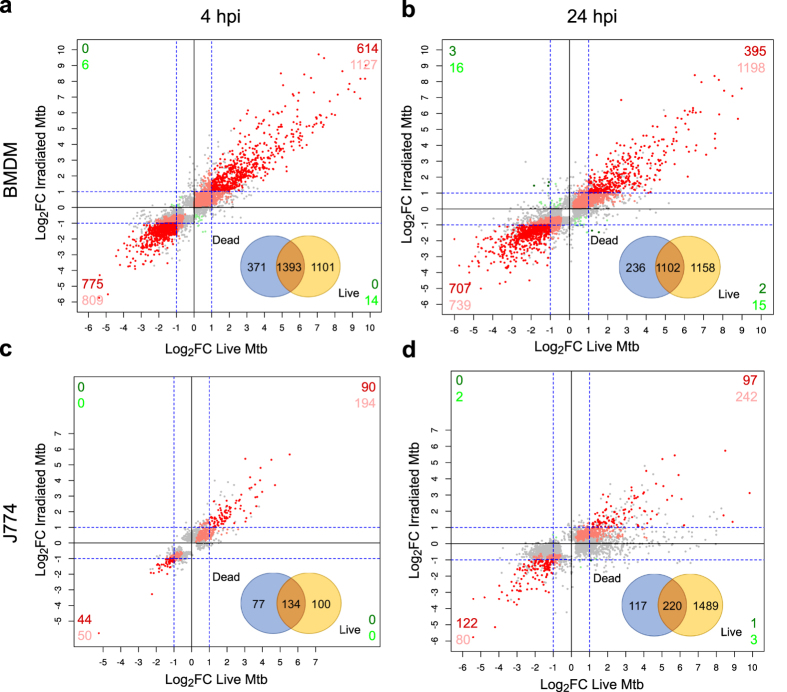
Comparison of the response of BMDMs and J774 to infection with live bacteria and stimulation with dead *M. tuberculosis*. Scatterplots comparing the Log_2_ fold change (FC) of all the differentially expressed genes (DEGs) in BMDMs at 4 hpi (**a**) and 24 hpi (**b**), and J774 at 4 hpi (**c**) and 24 hpi (**d**). Grey dots represent genes differentially expressed in one condition only. Points in shades of red represent genes differentially expressed in both data sets in the same direction. Points in shades of green represent genes differentially expressed in opposite direction. Those showing FC < |2| in either/both condition(s) are shown in light colour and those with FC > |2| in both conditions are shown in dark. The number of genes are indicated for each quadrant. A Venn diagram showing the number of DEGs in macrophages infected with live bacteria and/or stimulated with dead bacteria is shown for each macrophage type and time point.

**Figure 5:**
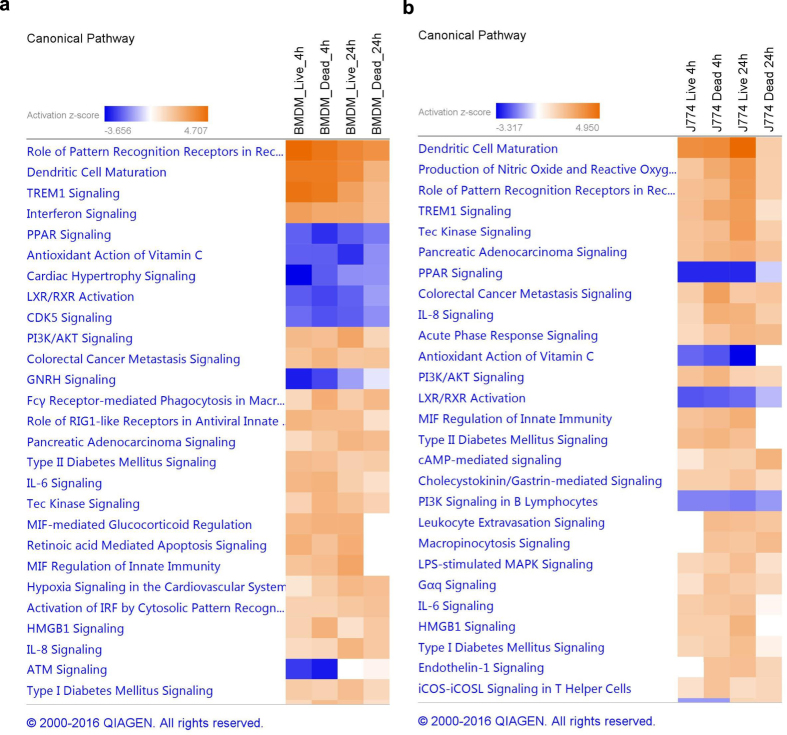
Top enriched pathways in macrophages infected with live bacteria and stimulated with dead *M. tuberculosis*. (**a**) BMDMs, and (**b**) J774 cells. Data were analysed through the use of QIAGEN’s Ingenuity^®^ Pathway Analysis (IPA^®^, QIAGEN Redwood City, www.qiagen.com/ingenuity). Pathways are ranked according to the *Z*-score that predicts activation/repression.

Pairwise comparisons of DEGs in macrophages stimulated with dead bacteria or infected with live bacteria showed a marked overlap, particularly in BMDM. 79% and 82% of the DEGs in BMDMs stimulated with dead bacteria were also differentially expressed in BMDMs infected with live bacteria at 4 hpi and 24 hpi, respectively (Fig. 4a and b). These correspond to 58% and 48% of the DEGs in BMDMs infected with live bacteria, respectively. Similarly, 64% and 65% of the DEGs in J774 stimulated with dead bacteria were also differentially expressed in J774 infected with live bacteria at 4 hpi and 24 hpi, respectively (Fig. 4c and d). These correspond to 57% of the DEGs in J774 infected with live bacteria at 4 hpi, but only 13% of the DEGs in J774 infected with live bacteria at 24 hpi (Fig. 4c and d). This reflects the substantial difference in the total number of DEGs between the two conditions in J774 at 24 hpi (Fig. 1b). Nevertheless, in all four comparisons virtually all the common genes were differentially expressed in the same direction and magnitude (Fig. 4). Consequently, pathway analysis results were similar in macrophages stimulated with dead bacteria and macrophages infected with live bacteria, and all the pathways had the same direction of activation/inhibition prediction (Fig. 5). As expected, the degree of activation/inhibition (*Z*-score absolute value) was similar in macrophages stimulated with dead bacteria or infected with live bacteria at 4 hpi but lower in macrophages stimulated with dead bacteria at 24 hpi. Overall, these results indicate that the differences detected in the transcriptomic responses between the two cell types occur even just upon phagocytosis of dead *M. tuberculosis*.

## Discussion

Macrophages play an essential role in the early immune response against *M. tuberculosis* and are the cell type preferentially infected *in vivo*. The failure of an appropriate macrophage response is critical for the establishment of infection and progression of the disease. *M. tuberculosis* has evolved multiple evasion mechanisms in order to survive and grow inside macrophages. To study the antimicrobial response of macrophages and the evasion mechanisms of *M. tuberculosis* both primary macrophages and cell lines are commonly used. BMDMs are a popular model because they are easy to obtain, and can be sourced from a variety of specific genetic backgrounds. Likewise, the cell line J774 is frequently used since it is readily available, and no animals are involved, which results in a reduction in the number of animals used in research. In the present study, we compare the transcriptomic response of BMDMs and the macrophage-like cell line J774 to infection with *M. tuberculosis* H37Rv by using high-throughput RNA-seq.

We have found that BMDMs respond very strongly to infection whereas the response of J774 to *M. tuberculosis* infection is weaker both in terms of number of DEGs and magnitude of induction/repression, at least during the early time points of the infection. A strong host response similar to that obtained in BMDMs has been previously observed using microarrays in murine BMDMs infected with live *M. tuberculosis* strains CDC1551 and HN878 by Koo *et al*.^42^. A high number of DEGs were detected early in the infection (6 hpi) followed by a decrease in the number of DEGs at 24 hpi^42^. Other studies using human or bovine MDMs have reported a more modest response in the first hours of the infection followed by a marked increase in the amount of DEGs detected at around 18–24 hpi, similar to what we have observed in the J774 cell line^43^,^44^,^45^. Interestingly, previous work has shown that infection with *Yersinia enterocolitica* results in a higher number of upregulated genes in BMDMs than in J774 cells^46^,^47^. This seems to be due to a higher susceptibility of J774 to inhibition by specific anti-host effector proteins from *Y. enterocolitica* that interfere with host signalling pathways^47^. Whether a similar scenario occurs upon infection with *M. tuberculosis* would be difficult to establish considering the multifactorial nature of its virulence. An additional explanation would be that J774, which derive from a lymphoma, are already in a relatively activated state with enhanced glycolysis, which may have contributed to the reduced response observed at 4 hpi compared to the more naïve BMDM.

It is important to note that the BMDMs used in the present study were obtained from C57BL/6 mice, an extensively studied mouse strain from which most knockout mutants derive, whereas the J774 cell line derives from Balb/c mice. Some differences have been described in the immune response of these two mouse strains to *M. tuberculosis* infection and BCG vaccination *in vivo*, entailing mainly a greater early innate response (measured by production of IL-12, IFN-γ, TNF-α, MCP-1 and neutrophil influx) but a suppressed Th1 response in Balb/c compared to C57BL/6^48^,^49^. However, both strains control the infection similarly and have been classified as resistant to *M. tuberculosis* infection in comparison to other more susceptible mouse strains^50^. Besides, they both mount a comparable protective immunity upon vaccination with BCG^51^. Additionally, their BMDMs respond very similarly to *M. tuberculosis* infection *in vitro*^52^,^53^. However, we cannot rule out that the differences we have observed are the result of both the different origin of the cells and of them being primary cells and a cell line.

Except for the more modest response of J774 cells, the type of response of both macrophage types to *M. tuberculosis* infection was quite similar in terms of DEGs and pathways enriched, particularly at 24 hpi. Importantly, several previously described antimycobacterial mechanisms were induced in our dataset such as *Irgm1 (Lrg-47*) and *Irgm2*, or *Isg15*. Many other ISGs with known antimicrobial function were also induced, such as *Rsad2 (Viperin*) or *Ifit1/2/3*. To the best of our knowledge, a specific role for many of these genes in *M. tuberculosis* infection has not been described. Therefore, the study of their involvement in the defence against *M. tuberculosis* is worth exploring.

An interesting pathway that was highly induced in both macrophages was TREM1. TREM1 has been shown to regulate the host response at an early stage during infection of various pathogens such as *Streptococcus pneumoniae*^54^, *Klebsiella pneumoniae*^55^, and *Streptococcus suis*^56^, and it is induced in humans during active tuberculosis^57^. Although the ligand of TREM1 is unknown, it has been speculated that pathogen-associated molecular patterns could directly activate it^31^, therefore it would be interesting to study if any of the *M. tuberculosis* cell wall components is a ligand for this receptor.

The PPAR signalling pathway was repressed. Current evidence indicates that mycobacterial infection causes a time-dependent increase in PPARγ expression through a macrophage mannose receptor-dependent pathway^58^. This induction results in increased lipid droplet formation and down-modulation of the macrophage response, suggesting that PPARγ expression might aid the mycobacteria in circumventing the host response. In fact, knockdown of PPARγ in human macrophages has been shown to result in an increase in TNF production and a decrease in *M. tuberculosis* growth at 24 hpi^58^. The inhibition of this pathway together with the antimicrobial mechanisms previously mentioned could explain the control of *M. tuberculosis* growth by the macrophage at this early stage of the infection^9^,^59^,^60^,^61^. PPAR is a well-known pathway associated with diabetes^62^. Interestingly there is now a recognised connection between diabetes and tuberculosis^63^, although the mechanisms leading to this interaction are still unclear^64^,^65^. However, with the PPARγ pathway common between these diseases, it could be worthwhile to explore this connection in more detail.

It has been recently recognised that *M. tuberculosis* derived-products reach the cytosol early during infection via the formation of pores in the phagosomal membrane in an ESX1-dependent process^66^. Both *M. tuberculosis* DNA and cyclic-di-adenosine monophosphate (c-di-AMP) are recognized by cytosolic receptors triggering production of type I IFN, ISGs and pro-inflammatory cytokines such as IL-1β through the (cGAS)-STING-TBK1-IRF3 and AIM2-NLRP3-IL-1β signalling pathways^67^,^68^. Surprisingly, the STING/TBK1 axis was not differentially expressed in our dataset but the genes for other DNA sensors such as *Dai (Zbp*) and *Ifi204*, and for RNA sensors such as RIG-I (*Ddx58*), LGP2 (*Dhx58*), and MDA5 (*Ifih1*) were induced, as were the downstream transcription factors NF-κB, IRF7, IRF9, STAT1 and STAT2, the effectors IFNβ and IL-1β, and many of the ISGs, particularly in BMDMs (at both time points, and either upon infection with live bacteria or stimulation with dead bacteria), but only marginally in J774 infected with live bacteria at 24 hpi. The importance of cytosolic nucleic acid receptors in the antimicrobial defence against bacteria has been increasingly recognized^69^,^70^. The relevance of the activation of these signalling pathways during *M. tuberculosis* infection and the origin of the DNA/RNA that they would sense deserves further investigation.

The similarity in the host response in macrophages infected with live bacteria or stimulated with γ-irradiated bacteria at this early stage of the infection is not surprising if we consider that, contrary to what happens with heat-killing, inactivation by γ-irradiation preserves cell integrity^71^. Therefore, the same pathogen associated molecular patterns (PAMPs) are recognized by the cell’s pattern recognition receptors (PRRs) leading to the same downstream signalling pathways being activated^72^. Furthermore, several studies have shown that cells inactivated by γ-irradiation are still metabolically active and *de novo* transcription and translation still occurs^71^,^73^. Consequently, it would be safe to speculate that during macrophage stimulation with γ-irradiated *M. tuberculosis* the early host-pathogen interactions are conserved. This would most likely include activation of the cytosolic surveillance pathway through ESX-1-mediated cytosolic access, in the same way that γ-irradiated *Francisella tularensis* is able to escape the endosome into the cytoplasm^74^. In fact, many of the hallmark genes of the cytosolic surveillance pathway were highly induced in BMDMs stimulated with dead bacteria. The induction was particularly high at 4 hpi and it decreased at 24 hpi (Log_2_FC ≈ 1–2) while it was still high in macrophages infected with live bacteria (Log_2_FC ≈ 3–4). It is likely that, at later time points, macrophages stimulated with inactivated bacteria incapable of replication can digest the bacteria and slowly shutdown their response. This would also explain the low number of DEGs at 24 hpi in macrophages stimulated with dead bacteria compared to macrophages infected with live bacteria.

In conclusion, comparison of the response of bone marrow-derived primary macrophages and the macrophage-like cell line J774 has highlighted differences in the timing and strength of the response. These differences should be taken into account when using a macrophage model for the study of host-pathogen interactions. Furthermore, the use of high-throughput RNA-seq has allowed delineation of the macrophage transcriptomic response to *M. tuberculosis* infection in great depth, identifying genes whose function in resistance to *M. tuberculosis* infection has not yet been investigated. These findings point to potentially novel processes leading to immune containment early in the infection.

## Methods

### Bacterial strains, growth, and γ-irradiation conditions

*M. tuberculosis* H37Rv (ATCC 27294) was grown in Middlebrook 7H9 supplemented with 0.05% Tween 80 and 10% albumin-dextrose-catalase (ADC), or on 7H11 agar supplemented with 10% oleic acid-albumin-dextrose-catalase (OADC) at 37 °C. Log-phase cultures were collected, washed twice with PBS + 0.05% Tween80 (PBS-T), and resuspended in Dulbecco’s PBS (DPBS) before measuring the absorbance at 600 nm and plating CFUs. 1 ml aliquots in 2 ml tubes were irradiated at room temperature at 2.5 × 10^4^ Gy using a Cs^137^ irradiator. Inactivation of the bacteria was verified by inoculating 100 μl in liquid and solid media and incubating for 6 weeks.

### Infection of BMDMs and J774 cells with *M. tuberculosis*

Animal protocols were performed in accordance with the Animals (Scientific Procedures) Act of 1986 and were approved by the animal welfare and ethical review board of the London School of Hygiene and Tropical Medicine. Bone marrow cells were flushed from the femur and tibia of 8 to 10 week old female C57BL/6 mice and differentiated into macrophages for 7 days in DMEM (Sigma) supplemented with 10% heat-inactivated fetal bovine serum (Biosera) and 20 ng/ml M-CSF (PeproTech). On day 4, cells were fed with an additional 10 ml of media. After 7 days in culture, cells were washed with DPBS and 5 × 10^6^ cells were seeded into 25 cm^2^ tissue culture flasks.

J774A.1 cells obtained from the ATCC (TIB-67) were cultured in DMEM (Sigma) supplemented with 10% heat-inactivated fetal bovine serum (Biosera) and 5 × 10^6^ cells were seeded into 25 cm^2^ tissue culture flasks for infection.

Mid-log phase *M. tuberculosis* were washed twice with PBS-T, once with DPBS, diluted in DMEM and added to either BMDMs or J774 cells at a concentration of ∼2.5 × 10^7^ CFU/flask (MOI of 5). After 4 h of infection at 37 °C in 5% CO_2_, macrophages were treated with 200 mg/l amikacin for 1 h and washed twice with DPBS to eliminate any extracellular bacteria. Lastly, 6 ml of complete DMEM (J774), or DMEM supplemented with 10 ng/ml M-CSF (BMDMs), was added to each flask. Cells were either immediately processed or incubated for further 20 hours. Infections were done in triplicate.

Intracellular survival and growth was assessed by lysis of the monolayers by the addition of 0.1% Triton X-100 in DPBS and enumeration of bacteria by serial dilution in PBS-T plating onto Middlebrook 7H11 solid medium. Colonies were counted after 3–4 weeks incubation at 37 °C and the average CFU/ml determined.

### RNA extraction

Macrophages were lysed by adding 4 M GTC solution (4 M guanidine thiocyanate, 0.5% sodium N–Lauroylsarcosine, 0.1 M β–mercaptoethanol, 0.5% Tween 80), and the lysate homogenized using a QIAshredder column (Qiagen). RNA was extracted by BCP phase partitioning and isopropanol precipitation, cleaned with 75% ethanol and treated with Turbo DNAse (Ambion, Life Technologies). DNA contamination was checked by PCR using primers for the RNA polymerase II gene *Rpb2* (Rpb2_Up: 5′ CTATAACCTGAATGTAGCAAGC 3′, Rpb2_Lw: 5′ CCCAATGAGGTGCTAGACTC 3′). The RNA was further cleaned using an RNeasy MinElute Cleanup kit (Qiagen), and its quality and quantity were determined using a Bioanalyzer (Agilent) and Qubit^®^ RNA HS kit (Invitrogen), respectively.

### Strand-specific RNA-seq library preparation and sequencing

Approximately 500 ng of RNA from each sample was used to prepare individually barcoded strand-specific RNA-seq libraries. rRNA and mitochondrial RNA was removed using Ribo-Zero Gold Removal Magnetic Kit for human/mouse/rat (Epicentre, now Illumina) following the manufacturer’s instructions. mRNA was purified using Agencourt RNAClean XP kit. Libraries were constructed using ScriptSeq™ Complete Kit (Epicentre, now Illumina) as per manufacturer’s instructions. Quality and quantity of the libraries were determined using a Bioanalyzer (Agilent) and Qubit^®^ (Invitrogen), respectively. Libraries were then quantified by qPCR in an Applied Biosystems^®^ 7500 with the Kapa Biosystems kit for library quantitation (Kapa Biosystems). Next-generation sequencing was performed using an Illumina HiSeq 2000 flow cell with one 150-bp end run. The NCBI Gene Expression Omnibus (GEO) accession number for the RNA-seq data reported in this paper is GSE88801.

### RNA-seq data analysis

Reads were aligned versus the Ensembl mouse genome version mm10 (GRCm38) using STAR^75^ 2.4.2a (Parameters: –outFilterMultimapNmax 20 –lnIntronMax 1000000 –alnSJoverhangMin 8 –alnSJDBoverhangMin 1). Uniquely aligned reads in BAM format were annotated against the protein-coding mRNA regions using SeqMonk v.0.33.1 platform (Babraham Bioinformatics, Cambridge UK). A median of 39.7 (31.7–59.3) and 51.3 (20.1–78.4) million single-end reads per library were obtained for the BMDMs and J774 libraries, respectively (Supplementary Data S5). Of these, a median of 82.47% (78.86–85.87%) and 78.93% (69.63–84.09%) of the reads mapped to unique locations in the *Mus musculus* reference genome (Supplementary Data S5, Figure S4), with a median of 92.25% (73.80–96.75%) and 93.88% (70.67–96.55%) assigned to genes (Supplementary Data S5, Figure S5). Contamination with rRNA or mitochondrial DNA was very low at a median of only 0.28% (0.15–4.47%) and 1.26% (0.95–5.34%), respectively, for the BMDMs libraries, and a 1.12% (0.07–4.69%) and 1.98% (0.71–5.28%) for the J774 libraries (Supplementary Data S5, Figure S5). Differential expression was analysed in SeqMonk after quantification of raw counts, strand-specific, merged isoforms, using the R (version 3.2.2) package DESeq2. A gene was classified as up- or downregulated using a cut-off value of more or less than 2-fold expression difference, with an adjusted *P*-value < 0.05, following multiple testing correction. DEGs were further analysed using QIAGEN’s Ingenuity^®^ Pathway Analysis (IPA^®^, QIAGEN) to identify over-represented canonical pathways.

## Additional Information

**How to cite this article:** Andreu, N. *et al*. Primary macrophages and J774 cells respond differently to infection with *Mycobacterium tuberculosis. Sci. Rep.* **7**, 42225; doi: 10.1038/srep42225 (2017).

**Publisher's note:** Springer Nature remains neutral with regard to jurisdictional claims in published maps and institutional affiliations.

## Supplementary Material

Supplementary Information

Supplementary Dataset S1

Supplementary Dataset S2

Supplementary Dataset S3

Supplementary Dataset S4

Supplementary Dataset S5

## References

[b1] Rajaram M. V., Ni B., Dodd C. E. & Schlesinger L. S. Macrophage immunoregulatory pathways in tuberculosis. Semin Immunol 26, 471–485 (2014). 25453226 10.1016/j.smim.2014.09.010 PMC4314327

[b2] Dey B. & Bishai W. R. Crosstalk between Mycobacterium tuberculosis and the host cell. Semin Immunol 26, 486–496 (2014). 25303934 10.1016/j.smim.2014.09.002 PMC4250340

[b3] Cadena A. M., Flynn J. L. & Fortune S. M. The Importance of First Impressions: Early Events in Mycobacterium tuberculosis Infection Influence Outcome. MBio 7 (2016). 10.1128/mBio.00342-16 PMC4817258 27048801

[b4] Johnson B. K. & Abramovitch R. B. Macrophage infection models for Mycobacterium tuberculosis. Methods Mol Biol 1285, 329–341 (2015). 25779326 10.1007/978-1-4939-2450-9_20

[b5] Mendoza-Coronel E. & Castanon-Arreola M. Comparative evaluation of *in vitro* human macrophage models for mycobacterial infection study. Pathog Dis 74, ftw052 (2016). 27307103 10.1093/femspd/ftw052

[b6] Burdall S. E., Hanby A. M., Lansdown M. R. J. & Speirs V. Breast cancer cell lines: friend or foe? Breast Cancer Research 5, 89–95 (2003). 12631387 10.1186/bcr577 PMC154155

[b7] Pan C., Kumar C., Bohl S., Klingmueller U. & Mann M. Comparative proteomic phenotyping of cell lines and primary cells to assess preservation of cell type-specific functions. Molecular & cellular proteomics: MCP 8, 443–450 (2009). 18952599 10.1074/mcp.M800258-MCP200 PMC2649808

[b8] Frattini A. . High variability of genomic instability and gene expression profiling in different HeLa clones. Scientific Reports 5, 15377 (2015). 26483214 10.1038/srep15377 PMC4613361

[b9] Jordao L., Bleck C. K., Mayorga L., Griffiths G. & Anes E. On the killing of mycobacteria by macrophages. Cell Microbiol 10, 529–548 (2008). 17986264 10.1111/j.1462-5822.2007.01067.x

[b10] Mehta P. K., King C. H., White E. H., Murtagh J. J. Jr. & Quinn F. D. Comparison of *in vitro* models for the study of Mycobacterium tuberculosis invasion and intracellular replication. Infect Immun 64, 2673–2679 (1996). 8698494 10.1128/iai.64.7.2673-2679.1996 PMC174125

[b11] Dillon L. A., Suresh R., Okrah K., Corrada Bravo H., Mosser D. M. & El-Sayed N. M. Simultaneous transcriptional profiling of *Leishmania major* and its murine macrophage host cell reveals insights into host-pathogen interactions. BMC Genomics 16, 1108 (2015). 26715493 10.1186/s12864-015-2237-2 PMC4696162

[b12] Fernandes M. C., Dillon L. A., Belew A. T., Bravo H. C., Mosser D. M. & El-Sayed N. M. Dual transcriptome profiling of *Leishmania*-infected human macrophages reveals distinct reprogramming signatures. mBio 7, e00027–16 (2016). 27165796 10.1128/mBio.00027-16 PMC4959658

[b13] Klug-Micu G. M. . CD40 ligand and interferon-gamma induce an antimicrobial response against Mycobacterium tuberculosis in human monocytes. Immunology 139, 121–128 (2013). 23289765 10.1111/imm.12062 PMC3634544

[b14] Singh S. B. . Human IRGM regulates autophagy and cell-autonomous immunity functions through mitochondria. Nat Cell Biol 12, 1154–1165 (2010). 21102437 10.1038/ncb2119 PMC2996476

[b15] Kim B. H. . A family of IFN-gamma-inducible 65-kD GTPases protects against bacterial infection. Science 332, 717–721 (2011). 21551061 10.1126/science.1201711

[b16] Bogunovic D. . Mycobacterial disease and impaired IFN-gamma immunity in humans with inherited ISG15 deficiency. Science 337, 1684–1688 (2012). 22859821 10.1126/science.1224026 PMC3507439

[b17] Michelucci A. . Immune-responsive gene 1 protein links metabolism to immunity by catalyzing itaconic acid production. Proc Natl Acad Sci USA 110, 7820–7825 (2013). 23610393 10.1073/pnas.1218599110 PMC3651434

[b18] Hall C. J. . Immunoresponsive gene 1 augments bactericidal activity of macrophage-lineage cells by regulating beta-oxidation-dependent mitochondrial ROS production. Cell Metab 18, 265–278 (2013). 23931757 10.1016/j.cmet.2013.06.018

[b19] Roca F. J. & Ramakrishnan L. TNF dually mediates resistance and susceptibility to mycobacteria via mitochondrial reactive oxygen species. Cell 153, 521–534 (2013). 23582643 10.1016/j.cell.2013.03.022 PMC3790588

[b20] Krishnan N., Robertson B. D. & Thwaites G. Pathways of IL-1beta secretion by macrophages infected with clinical Mycobacterium tuberculosis strains. Tuberculosis (Edinb) 93, 538–547 (2013). 23849220 10.1016/j.tube.2013.05.002 PMC3759846

[b21] Jayaraman P. . IL-1beta promotes antimicrobial immunity in macrophages by regulating TNFR signaling and caspase-3 activation. J Immunol 190, 4196–4204 (2013). 23487424 10.4049/jimmunol.1202688 PMC3622150

[b22] Fantuzzi G. & Dinarello C. A. Interleukin-18 and interleukin-1 beta: two cytokine substrates for ICE (caspase-1). J Clin Immunol 19, 1–11 (1999). 10080100 10.1023/a:1020506300324

[b23] Volkman H. E. . Tuberculous granuloma induction via interaction of a bacterial secreted protein with host epithelium. Science 327, 466–469 (2010). 20007864 10.1126/science.1179663 PMC3125975

[b24] Vesosky B., Rottinghaus E. K., Stromberg P., Turner J. & Beamer G. CCL5 participates in early protection against Mycobacterium tuberculosis. J Leukoc Biol 87, 1153–1165 (2010). 20371596 10.1189/jlb.1109742 PMC2872537

[b25] Diamond M. S. IFIT1: A dual sensor and effector molecule that detects non-2′-O methylated viral RNA and inhibits its translation. Cytokine & growth factor reviews 25, 543–550 (2014). 24909568 10.1016/j.cytogfr.2014.05.002 PMC4234691

[b26] Haller O., Staeheli P., Schwemmle M. & Kochs G. Mx GTPases: dynamin-like antiviral machines of innate immunity. Trends in microbiology 23, 154–163 (2015). 25572883 10.1016/j.tim.2014.12.003

[b27] Helbig K. J. & Beard M. R. The role of viperin in the innate antiviral response. Journal of molecular biology 426, 1210–1219 (2014). 24157441 10.1016/j.jmb.2013.10.019

[b28] Khounlotham M., Subbian S., Smith R. 3rd, Cirillo S. L. & Cirillo J. D. Mycobacterium tuberculosis interferes with the response to infection by inducing the host EphA2 receptor. J Infect Dis 199, 1797–1806 (2009). 19426113 10.1086/599096 PMC4154492

[b29] Jager E. . Dendritic Cells Regulate GPR34 through Mitogenic Signals and Undergo Apoptosis in Its Absence. J Immunol 196, 2504–2513 (2016). 26851221 10.4049/jimmunol.1501326

[b30] Liebscher I. . Altered immune response in mice deficient for the G protein-coupled receptor GPR34. J Biol Chem 286, 2101–2110 (2011). 21097509 10.1074/jbc.M110.196659 PMC3023507

[b31] Arts R. J., Joosten L. A., van der Meer J. W. & Netea M. G. TREM-1: intracellular signaling pathways and interaction with pattern recognition receptors. J Leukoc Biol 93, 209–215 (2013). 23108097 10.1189/jlb.0312145

[b32] Wu M. . TREM-1 amplifies corneal inflammation after Pseudomonas aeruginosa infection by modulating Toll-like receptor signaling and Th1/Th2-type immune responses. Infect Immun 79, 2709–2716 (2011). 21555403 10.1128/IAI.00144-11 PMC3191966

[b33] Sathyamoorthy T. . Membrane Type 1 Matrix Metalloproteinase Regulates Monocyte Migration and Collagen Destruction in Tuberculosis. J Immunol 195, 882–891 (2015). 26091717 10.4049/jimmunol.1403110 PMC4505956

[b34] Schroder W. A. . A physiological function of inflammation-associated SerpinB2 is regulation of adaptive immunity. J Immunol 184, 2663–2670 (2010). 20130210 10.4049/jimmunol.0902187

[b35] Bufe B. . Recognition of bacterial signal peptides by mammalian formyl peptide receptors: a new mechanism for sensing pathogens. J Biol Chem 290, 7369–7387 (2015). 25605714 10.1074/jbc.M114.626747 PMC4367248

[b36] Hu X. . Integrated regulation of Toll-like receptor responses by Notch and interferon-gamma pathways. Immunity 29, 691–703 (2008). 18976936 10.1016/j.immuni.2008.08.016 PMC2585039

[b37] Strick D. J. & Elferink L. A. Rab15 effector protein: a novel protein for receptor recycling from the endocytic recycling compartment. Mol Biol Cell 16, 5699–5709 (2005). 16195351 10.1091/mbc.E05-03-0204 PMC1289414

[b38] Zuk P. A. & Elferink L. A. Rab15 differentially regulates early endocytic trafficking. J Biol Chem 275, 26754–26764 (2000). 10837464 10.1074/jbc.M000344200

[b39] Kruidenier L. . A selective jumonji H3K27 demethylase inhibitor modulates the proinflammatory macrophage response. Nature 488, 404–408 (2012). 22842901 10.1038/nature11262 PMC4691848

[b40] Holla S. . MUSASHI-Mediated Expression of JMJD3, a H3K27me3 Demethylase, Is Involved in Foamy Macrophage Generation during Mycobacterial Infection. PLoS Pathog 12, e1005814 (2016). 27532872 10.1371/journal.ppat.1005814 PMC4988650

[b41] Khader S. A. . IL-23 is required for long-term control of Mycobacterium tuberculosis and B cell follicle formation in the infected lung. J Immunol 187, 5402–5407 (2011). 22003199 10.4049/jimmunol.1101377 PMC3208087

[b42] Koo M. S., Subbian S. & Kaplan G. Strain specific transcriptional response in Mycobacterium tuberculosis infected macrophages. Cell Commun Signal 10, 2 (2012). 22280836 10.1186/1478-811X-10-2 PMC3317440

[b43] Blischak J. D., Tailleux L., Mitrano A., Barreiro L. B. & Gilad Y. Mycobacterial infection induces a specific human innate immune response. Sci Rep 5, 16882 (2015). 26586179 10.1038/srep16882 PMC4653619

[b44] Nalpas N. C. . RNA sequencing provides exquisite insight into the manipulation of the alveolar macrophage by tubercle bacilli. Sci Rep 5, 13629 (2015). 26346536 10.1038/srep13629 PMC4642568

[b45] Tailleux L. . Probing host pathogen cross-talk by transcriptional profiling of both Mycobacterium tuberculosis and infected human dendritic cells and macrophages. PLoS One 3, e1403 (2008). 18167562 10.1371/journal.pone.0001403 PMC2151136

[b46] Hoffmann R., Van Erp K., Trülzsch K. & Heesemann J. Transcriptional responses of murine macrophages to infection with Yersinia enterocolitica. Cellular Microbiology 6, 377–390 (2004). 15009029 10.1111/j.1462-5822.2004.00365.x

[b47] van Erp K., Dach K., Koch I., Heesemann J. & Hoffmann R. div xmlns= http://www.w3.org/1999/xhtml Role of strain differences on host resistance and the transcriptional response of macrophages to infection with <em >Yersinia enterocolitica </em> </div>. Physiological Genomics 25, 75–84 (2006). 16352694 10.1152/physiolgenomics.00188.2005

[b48] Jung Y. J., Ryan L., LaCourse R. & North Robert J. Differences in the Ability to Generate Type 1 T Helper Cells Need Not Determine Differences in the Ability to Resist Mycobacterium tuberculosis Infection among Mouse Strains. Journal of Infectious Diseases 199, 1790–1796 (2009). 19426112 10.1086/599092

[b49] Wakeham J., Wang J. & Xing Z. Genetically Determined Disparate Innate and Adaptive Cell-Mediated Immune Responses to Pulmonary Mycobacterium bovis BCG Infection in C57BL/6 and BALB/c Mice. Infection and Immunity 68, 6946–6953 (2000). 11083818 10.1128/iai.68.12.6946-6953.2000 PMC97803

[b50] Beamer G. L. & Turner J. Murine models of susceptibility to tuberculosis. Arch Immunol Ther Exp (Warsz) 53, 469–483 (2005). 16407780

[b51] Garcia-Pelayo M. C., Bachy V. S., Kaveh D. A. & Hogarth P. J. BALB/c mice display more enhanced BCG vaccine induced Th1 and Th17 response than C57BL/6 mice but have equivalent protection. Tuberculosis 95, 48–53 (2015). 25467292 10.1016/j.tube.2014.10.012

[b52] Keller C., Lauber J., Blumenthal A., Buer J. & Ehlers S. Resistance and susceptibility to tuberculosis analysed at the transcriptome level: lessons from mouse macrophages. Tuberculosis (Edinb) 84, 144–158 (2004). 15207484 10.1016/j.tube.2003.12.003

[b53] Lee H.-J., Ko H.-J. & Jung Y.-J. Insufficient Generation of Mycobactericidal Mediators and Inadequate Level of Phagosomal Maturation Are Related with Susceptibility to Virulent Mycobacterium tuberculosis Infection in Mouse Macrophages. Frontiers in Microbiology 7 (2016). 10.3389/fmicb.2016.00541 PMC4834433 27148227

[b54] Hommes T. J. . Triggering receptor expressed on myeloid cells-1 (TREM-1) improves host defence in pneumococcal pneumonia. J Pathol 233, 357–367 (2014). 24752755 10.1002/path.4361

[b55] Hommes T. J. . Role of triggering receptor expressed on myeloid cells-1/3 in Klebsiella-derived pneumosepsis. Am J Respir Cell Mol Biol 53, 647–655 (2015). 25860078 10.1165/rcmb.2014-0485OC

[b56] Yang C. . TREM-1 signaling promotes host defense during the early stage of infection with highly pathogenic Streptococcus suis. Infect Immun 83, 3293–3301 (2015). 26056380 10.1128/IAI.00440-15 PMC4496610

[b57] Joosten S. A., Fletcher H. A. & Ottenhoff T. H. A helicopter perspective on TB biomarkers: pathway and process based analysis of gene expression data provides new insight into TB pathogenesis. PLoS One 8, e73230 (2013). 24066041 10.1371/journal.pone.0073230 PMC3774688

[b58] Rajaram M. V. . Mycobacterium tuberculosis activates human macrophage peroxisome proliferator-activated receptor gamma linking mannose receptor recognition to regulation of immune responses. J Immunol 185, 929–942 (2010). 20554962 10.4049/jimmunol.1000866 PMC3014549

[b59] Pethe K. . Isolation of Mycobacterium tuberculosis mutants defective in the arrest of phagosome maturation. Proc Natl Acad Sci USA 101, 13642–13647 (2004). 15340136 10.1073/pnas.0401657101 PMC518761

[b60] Homolka S., Niemann S., Russell D. G. & Rohde K. H. Functional genetic diversity among Mycobacterium tuberculosis complex clinical isolates: delineation of conserved core and lineage-specific transcriptomes during intracellular survival. PLoS Pathog 6, e1000988 (2010). 20628579 10.1371/journal.ppat.1000988 PMC2900310

[b61] Rohde K. H., Veiga D. F., Caldwell S., Balazsi G. & Russell D. G. Linking the transcriptional profiles and the physiological states of Mycobacterium tuberculosis during an extended intracellular infection. PLoS Pathog 8, e1002769 (2012). 22737072 10.1371/journal.ppat.1002769 PMC3380936

[b62] Ahmadian M. . PPARgamma signaling and metabolism: the good, the bad and the future. Nat Med 19, 557–566 (2013). 23652116 10.1038/nm.3159 PMC3870016

[b63] Pizzol D. . Tuberculosis and diabetes: current state and future perspectives. Tropical medicine & international health: TM & IH 21, 694–702 (2016). 27102229 10.1111/tmi.12704

[b64] Martinez N., Ketheesan N., West K., Vallerskog T. & Kornfeld H. Impaired Recognition of Mycobacterium tuberculosis by Alveolar Macrophages from Diabetic Mice. J Infect Dis (2016). 10.1093/infdis/jiw436 PMC5144731 27630197

[b65] Lachmandas E. . Diabetes Mellitus and Increased Tuberculosis Susceptibility: The Role of Short-Chain Fatty Acids. Journal of diabetes research 2016, 6014631 (2016). 27057552 10.1155/2016/6014631 PMC4709651

[b66] Simeone R. . Phagosomal rupture by Mycobacterium tuberculosis results in toxicity and host cell death. PLoS Pathog 8, e1002507 (2012). 22319448 10.1371/journal.ppat.1002507 PMC3271072

[b67] Dey B. . A bacterial cyclic dinucleotide activates the cytosolic surveillance pathway and mediates innate resistance to tuberculosis. Nat Med 21, 401–406 (2015). 25730264 10.1038/nm.3813 PMC4390473

[b68] Wassermann R. . Mycobacterium tuberculosis Differentially Activates cGAS- and Inflammasome-Dependent Intracellular Immune Responses through ESX-1. Cell Host Microbe 17, 799–810 (2015). 26048138 10.1016/j.chom.2015.05.003

[b69] Eigenbrod T. & Dalpke A. H. Bacterial RNA: An Underestimated Stimulus for Innate Immune Responses. The Journal of Immunology 195, 411–418 (2015). 26138638 10.4049/jimmunol.1500530

[b70] Patel J. R. & García-Sastre A. Activation and regulation of pathogen sensor RIG-I. Cytokine & growth factor reviews 25, 513–523 (2014). 25212896 10.1016/j.cytogfr.2014.08.005

[b71] Secanella-Fandos S., Noguera-Ortega E., Olivares F., Luquin M. & Julian E. Killed but metabolically active Mycobacterium bovis bacillus Calmette-Guerin retains the antitumor ability of live bacillus Calmette-Guerin. The Journal of urology 191, 1422–1428 (2014). 24333111 10.1016/j.juro.2013.12.002

[b72] Datta S. K. . Vaccination with irradiated Listeria induces protective T cell immunity. Immunity 25, 143–152 (2006). 16860763 10.1016/j.immuni.2006.05.013

[b73] Magnani D. M., Harms J. S., Durward M. A. & Splitter G. A. Nondividing but metabolically active gamma-irradiated Brucella melitensis is protective against virulent B. melitensis challenge in mice. Infect Immun 77, 5181–5189 (2009). 19703982 10.1128/IAI.00231-09 PMC2772552

[b74] Bauler T. J., Chase J. C., Wehrly T. D. & Bosio C. M. Virulent Francisella tularensis destabilize host mRNA to rapidly suppress inflammation. J Innate Immun 6, 793–805 (2014). 24902499 10.1159/000363243 PMC4201887

[b75] Dobin A. . STAR: ultrafast universal RNA-seq aligner. Bioinformatics 29, 15–21 (2013). 23104886 10.1093/bioinformatics/bts635 PMC3530905

