# Supplementary material for: Primary macrophages and J774 cells respond differently to infection with Mycobacterium tuberculosis
Source: Sci Rep. 2017 Feb 8;7:42225. doi: 10.1038/srep42225 (PMC5296737; doi:10.1038/srep42225)

Primary macrophages and J774 cells respond differently to infection with *Mycobacterium tuberculosis*

Nuria Andreu[1,*], Jody Phelan[1], Paola F. de Sessions[2], Jacqueline M. Cliff[3], Taane G. Clark[1,4], and Martin L. Hibberd[1,2,*]

[1]Pathogen Molecular Biology Department, London School of Hygiene and Tropical Medicine, London, UK

[2]Genome Institute of Singapore, A*STAR, Singapore, Singapore

[3]TB Centre and Department of Immunology and Infection, London School of Hygiene and Tropical Medicine, London, UK

[4]Department of Infectious Disease Epidemiology, London School of Hygiene and Tropical Medicine, London, UK

*Corresponding authors: nuria.andreum@gmail.com, Martin.Hibberd@lshtm.ac.uk

Supplementary Figure S1. Infection of BMDMs and J774 with *M. tuberculosis*. Cells (5x10^{6}) were infected with live *M. tuberculosis* at an MOI of 5. CFUs were plated from lysed macrophages at 4 and 24 hpi. No significant differences were detected in the bacterial load in the two types of cells.

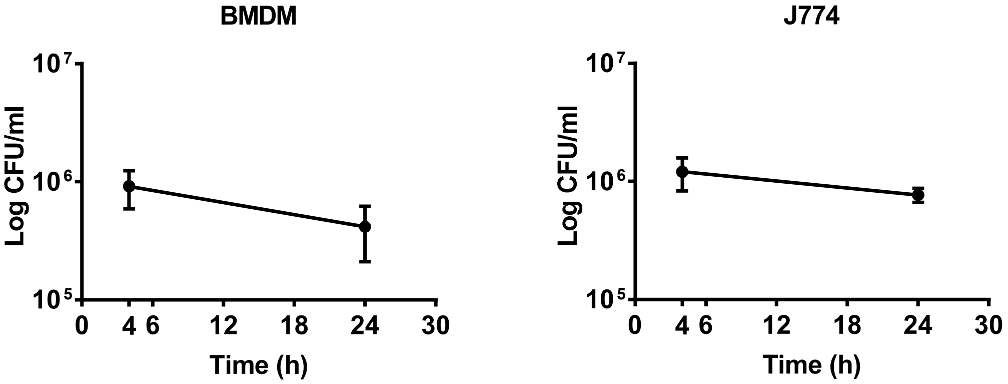

Supplementary Figure S2. Global expression profiles of macrophages during *M. tuberculosis* infection. PCA was conducted to evaluate the relationship between samples across time points for BMDMs and J774 cells. Each dot represents an experimental sample. Colours indicate sample type (shown in colour key). B: BMDMs, J: J774, U: Uninfected, I: γ-Irradiated *M. tuberculosis*-stimulated, L: Live *M. tuberculosis*-infected, 4: 4 hpi, 24: 24 hpi.

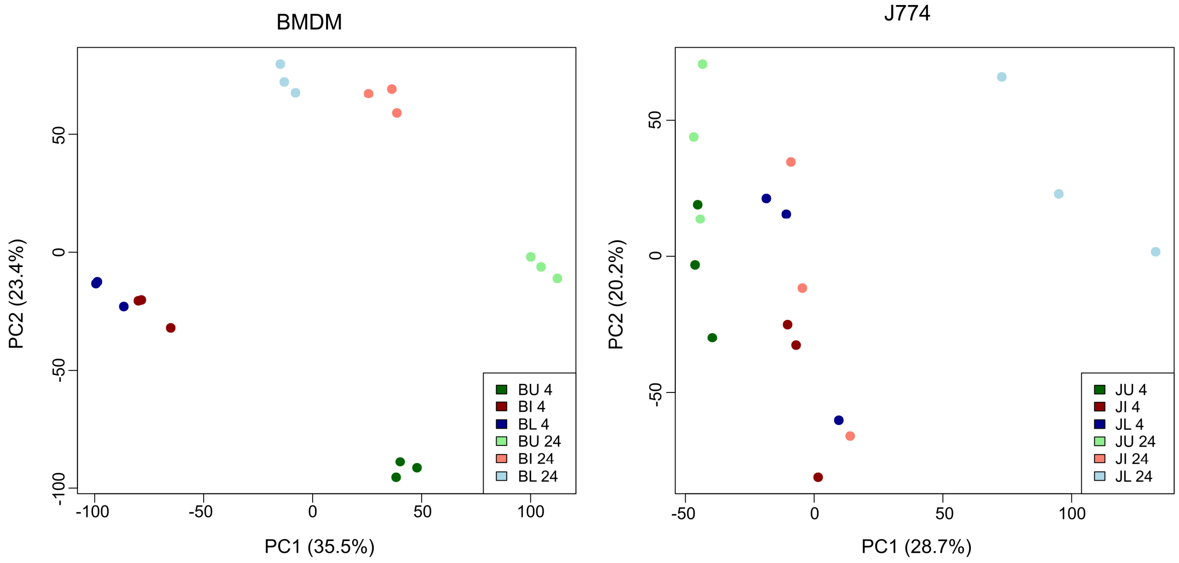

Supplementary Figure S3. TREM-1 signalling pathway. **a)** The TREM-1 signalling pathway is represented with gene expression (Log_2FC) values of BMDMs infected with *M. tuberculosis* versus the uninfected controls overlaid. The colour intensity corresponds to the level of upregulation (red) or downregulation (green). Genes coloured in grey were not significantly differentially expressed or did not pass the $|Log_2FC|>1$ cut-off. **b)** Heat map showing the gene expression values (Log_2FC) for all the genes of the TREM-1 signalling pathway in BMDMs and J774 infected with live *M. tuberculosis* at 4 and 24 hpi. The network and heat map were generated through the use of QIAGEN's Ingenuity® Pathway Analysis (IPA®, QIAGEN Redwood City, www.qiagen.com/ingenuity).

a

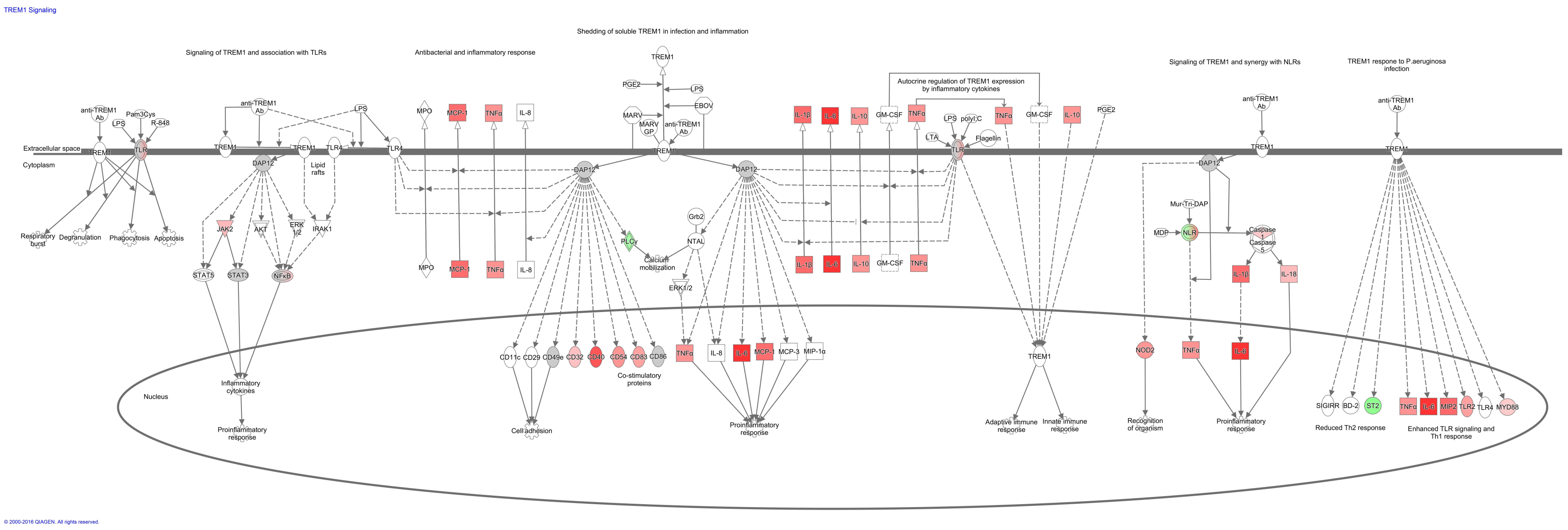

b

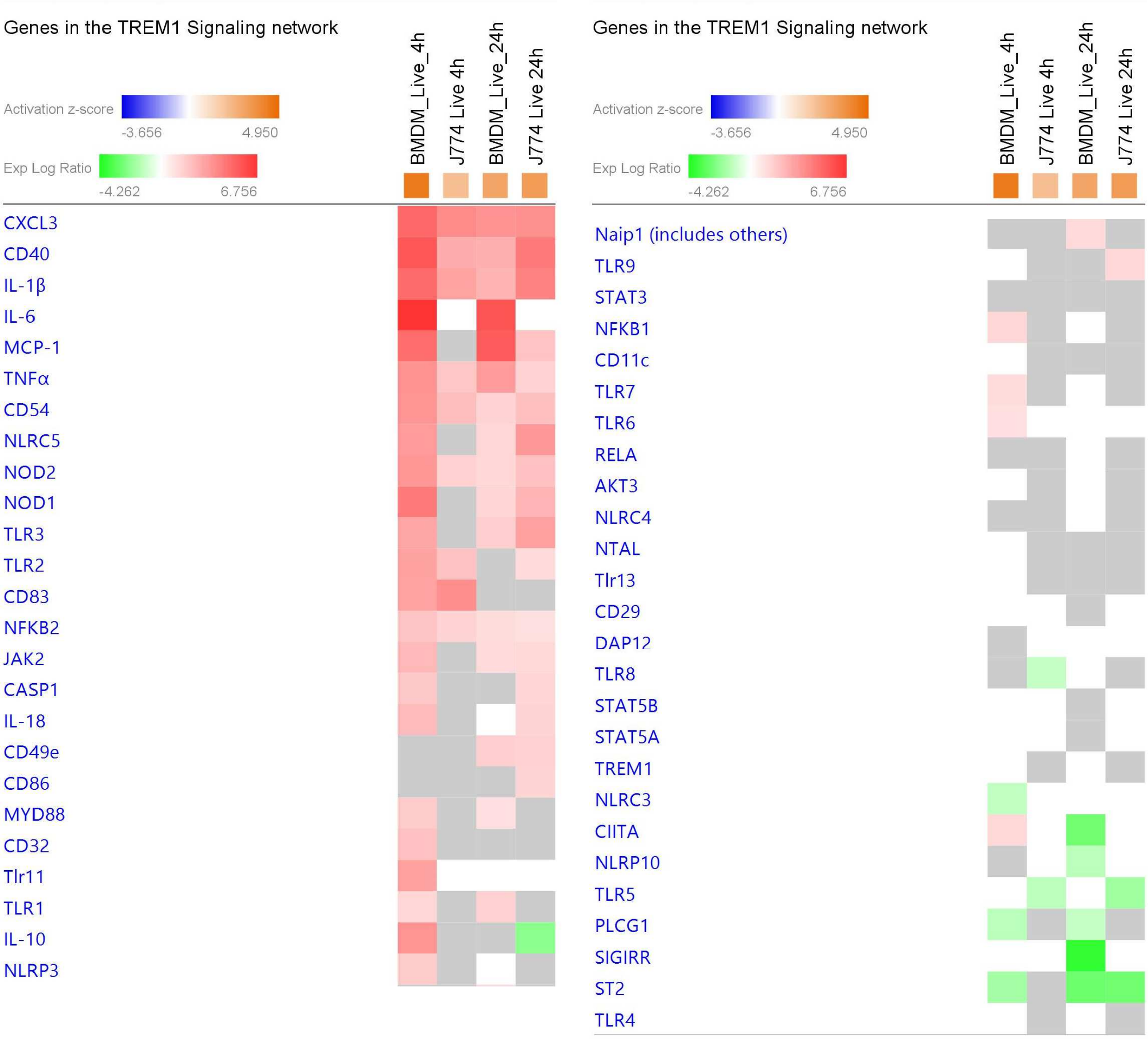

© 2000-2016 QIAGEN. All rights reserved.

Supplementary Figure S4. Libraries alignment statistics. “Unmapped” refers to reads that did not map to the mouse genome. “Uniquely mapped”, refers to reads that mapped to a unique site in the genome, whereas “mapped” refers to reads that mapped to multiple loci. B: BMDMs, J: J774, U: Uninfected, I: γ-Irradiated *M. tuberculosis*-stimulated, L: Live *M. tuberculosis*-infected, 4: 4 hpi, 24: 24 hpi.

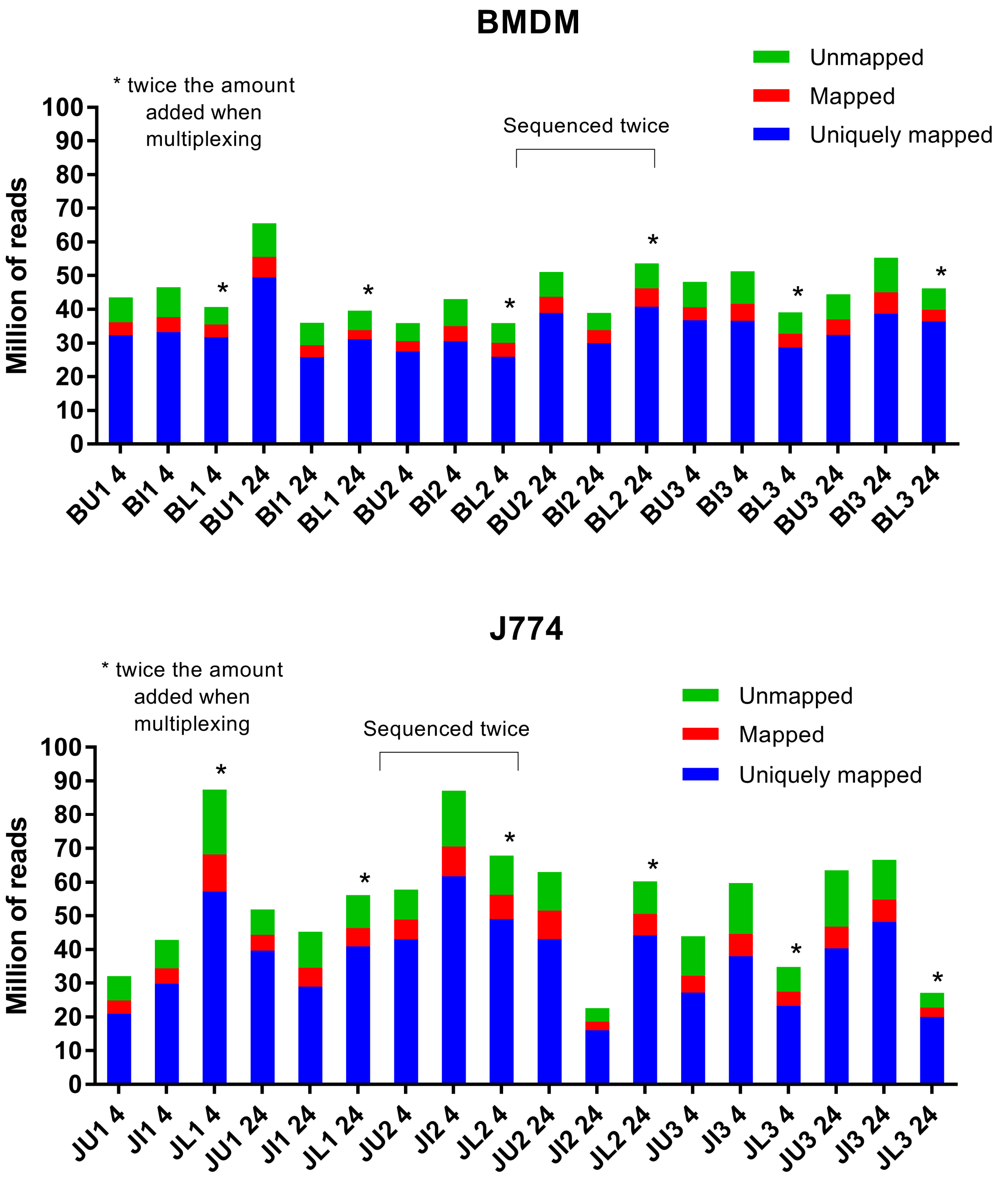

Supplementary Figure S5. Libraries mapping statistics. The percentage of reads mapped to exons (including mitochondrial DNA), introns, intergenic regions and rRNA is shown. B: BMDMs, J: J774, U: Uninfected I: γ-Irradiated *M. tuberculosis*-stimulated, L: Live *M. tuberculosis*-infected,4: 4 hpi, 24: 24 hpi.

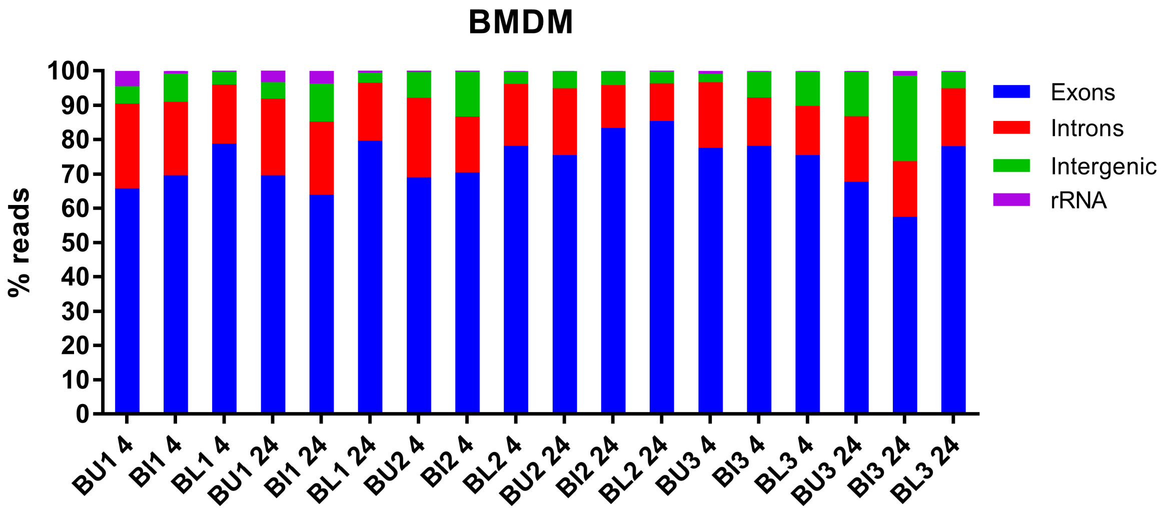

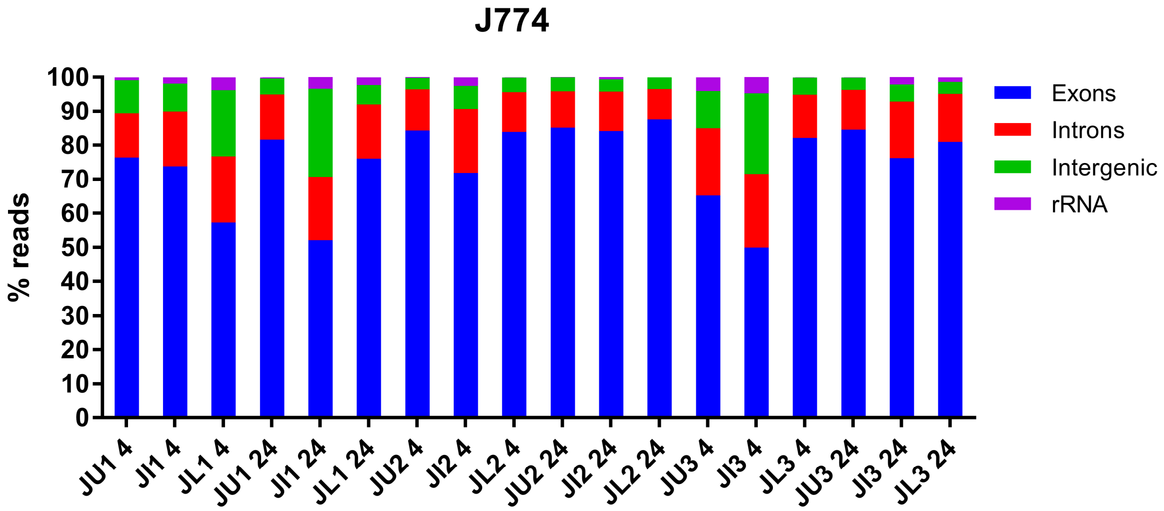

Supplement: Supplementary Information [file srep42225-s1.pdf]
